# Long-Term Acclimation to Different Thermal Regimes Affects Molecular Responses to Heat Stress in a Freshwater Clam *Corbicula Fluminea*

**DOI:** 10.1038/srep39476

**Published:** 2016-12-20

**Authors:** Halina I. Falfushynska, Tuan Phan, Inna M. Sokolova

**Affiliations:** 1 Department of Biological Sciences, University of North Carolina at Charlotte, 9201 University City Blvd., Charlotte, NC, USA; 2 I.Ya. Horbachevsky Ternopil State Medical University, Ternopil, Ukraine; 3 Department of Marine Biology, Institute of Biological Sciences, University of Rostock, Rostock, Germany

## Abstract

Global climate change (GCC) can negatively affect freshwater ecosystems. However, the degree to which freshwater populations can acclimate to long-term warming and the underlying molecular mechanisms are not yet fully understood. We used the cooling water discharge (CWD) area of a power plant as a model for long-term warming. Survival and molecular stress responses (expression of molecular chaperones, antioxidants, bioenergetic and protein synthesis biomarkers) to experimental warming (20–41 °C, +1.5 °C per day) were assessed in invasive clams *Corbicula fluminea* from two pristine populations and a CWD population. CWD clams had considerably higher (by ~8–12 °C) lethal temperature thresholds than clams from the pristine areas. High thermal tolerance of CWD clams was associated with overexpression of heat shock proteins HSP70, HSP90 and HSP60 and activation of protein synthesis at 38 °C. Heat shock response was prioritized over the oxidative stress response resulting in accumulation of oxidative lesions and ubiquitinated proteins during heat stress in CWD clams. Future studies should determine whether the increase in thermal tolerance in CWD clams are due to genetic adaptation and/or phenotypic plasticity. Overall, our findings indicate that *C. fluminea* has potential to survive and increase its invasive range during warming such as expected during GCC.

Freshwater ecosystems play a critical role in sustaining life and support a disproportionately high fraction of the Earth’s biodiversity. Because of their extensive use and the landlocked position, the freshwater ecosystems are among the most threatened habitats due to pollution, alterations in land and water use and global climate change^1^. Global climate change (GCC) is an imminent threat for freshwater ecosystems leading to warming, higher amplitude of thermal fluctuations and increased frequency of extreme weather events such as droughts and heat waves. The consensus scenarios of the Intergovernmental Panel for Climate Change (IPCC) forecast a 1.5–6 °C increase in the mean temperature worldwide (depending on the CO_2_ emission scenarios), with freshwater bodies warming significantly faster than most terrestrial and marine habitats^2^. This change can strongly affect freshwater organisms, which are >99% comprised of ectotherms and thus are sensitive to the direct effects of temperature on their physiological and biochemical processes.

Understanding the physiological and molecular mechanisms that limit species’ thermal tolerance in the face of GCC is critical for predicting the potential effects of GCC on aquatic populations^3^. An important challenge for the development of such physiologically-based mechanistic models is choosing a tractable experimental system that approximates the magnitude and the time scale of warming typical of GCC. Macrophysiological approaches comparing populations from different latitudes provide important insights into the mechanisms involved in the long-term acclimation or adaptation to different thermal regimes; however, such adaptations typically occur on much longer time scale than GCC and may be confounded by different evolutionary history of the populations from different latitudes^4^,^5^. On the other end of the spectrum, laboratory studies involving short-term (days to months) acclimations to different thermal regimes may underestimate the long-term effects and/or overestimate the thermal tolerance limits of aquatic organisms facing GCC^6^. Studies of the model populations sharing a recent common origin and exposed to long-term warming are therefore important to complement and advance our understanding of the potential effects of GCC on aquatic organisms. Cooling water discharge (CWD) areas of electrical power plants can serve as incidental field laboratories to study the effects of long-term warming. The CWD areas are characterized by distinctive thermal regimes with temperatures stably elevated above similar natural reservoirs permitting long-term acclimation of the resident biota^7^,^8^. Furthermore, long-term exposure to elevated temperatures in the CWD area occurs in the natural settings that preserve the essential habitat features and species interactions. This makes CWD areas experimental goldmines to study the responses of freshwater organisms to long-term warming in the field.

Freshwater bivalves such as clams and mussels are keystone organisms in freshwater ecosystems playing a key role in the food webs, sediment bioturbation and top-down control of water quality^9^,^10^. Due to their sedentary life style and filter-feeding behavior, freshwater bivalves can serve as bioindicators of the water quality and ecosystem health. The Asian clam *Corbicula fluminea* (Müller, 1774) is a common freshwater bivalve in the United States. It is an invasive species introduced from Asia around 1938 and spread throughout all the major watersheds of both Americas^11^,^12^. *C. fluminea* is a nuisance species that colonizes the pipes of water treatment systems and power stations altering flow and increasing sedimentation rates. Established *C. fluminea* populations also affect the water quality and sediment mixing through active filtration and burrowing behavior^13^,^14^. Due to their broad distribution, important role in freshwater ecosystems and common occurrence in the CWD areas of the power plants, *C. fluminea* are useful organisms to study the acclimation capacity to long-term warming. In this study, we focused on three populations of *C. fluminea* from two pristine areas (Lake Norman State Park, or LN, and Catawba River, or CR) and the water discharge channel of the Catawba Nuclear Station (CWD) in North Carolina, USA. Catawba Nuclear Station began commercial operations in 1985, approximately 15 years after *C. fluminea* colonized Catawba river^11^ so that the CWD population of *C. fluminea* was exposed to elevated temperatures near the power station outflow for ~30 years.

To gain insights into the acclimation potential of *C. fluminea* facing long-term warming, we determined thermal tolerance limits of clams from the three populations (LN, CR and CWD) and assessed their molecular stress responses and bioenergetic capacity during heat stress. We hypothesized that acclimation in the CWD area will result in an upward shift of the thermal tolerance limits of clams, and this shift will be associated with upregulation of stress protection mechanisms at high temperatures. To test these hypotheses, we determined the expression of stress-related genes (including heat shock proteins and antioxidants) and measured levels of oxidative lesions and protein damage during acute temperature rise from 20 to 41 °C (at a rate of 1.5 °C day^−1^) in *C. fluminea* from different habitats. We also assessed whether interpopulational differences in heat tolerance are associated with differential ability to maintain energy homeostasis during heat stress. Cellular energy status was assessed by expression of a key energy sensor, AMP-dependent protein kinase α (AMPKα) (Hardie, 2014). Transition to partial anaerobiosis (indicative of insufficient aerobic capacity) was determined by the relative activities of key enzymes at aerobic/anaerobic branchpoint [pyruvate kinase (PK), phosphoenolpyruvate carboxykinase (PEPCK), and lactate dehydrogenase (LDH)], and by stabilization of the hypoxia-inducible factor 1α (HIF-1α)^15^,^16^,^17^,^18^,^19^. Effects of acute warming on the protein synthesis was determined by measuring the expression of a key regulator of protein translation, the eukaryotic initiation factor eIF-2α (Larade and Storey, 2002; 2007). This comprehensive assessment of molecular and cellular stress responses provides insights into the mechanisms involved in the heat tolerance of clams exposed to chronically elevated temperatures.

## Results

### Heat tolerance

**Figure 1:**
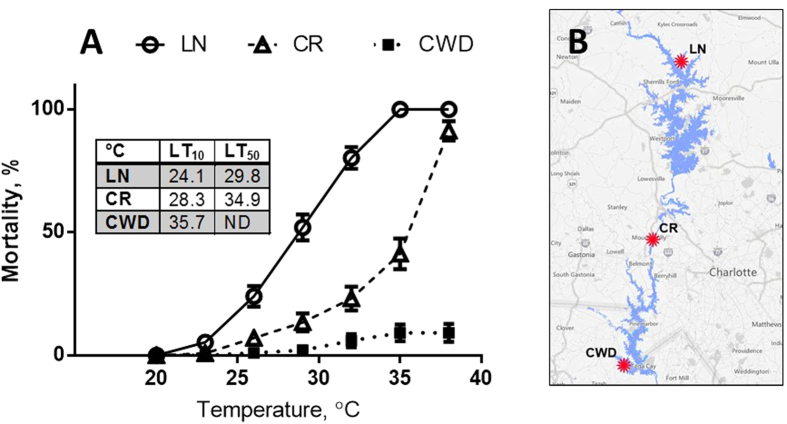
Mortality of *C. fluminea* from the three studied sites during acute temperature rise. (**A**) – mortality, (**B**) – geographic position of the studied C. fluminea populations in the Catawba River basin, map data: © 2016 Google. Populations: LN - Lake Norman site, CR – Catawba River site, CWD – cooling water discharge site. Temperature was raised at a rate of 1 °C per hour for 3 hours, followed by a 48 h acclimation period after every 3 °C rise. LT_10_ and LT_50_ are the temperatures causing 10% and 50% mortality in the studied populations, respectively. LT_50_ could not be determined in the CWD clams due to the low mortality rates at the temperatures ≤38 °C and 100% mortality at 41 °C. Vertical bars represent the standard error of estimate. N = 122 for LN and CR, and 119 for CWD.

Clams from the three studied populations differed in tolerance to the experimental temperature rise (+1.5 °C per day), with CWD clams being the most tolerant and those from the LN site – most susceptible to heat stress (Fig. 1). Thus, >90% of CWD clams survived 48 h of exposure to 38 °C, compared to only 10% of CR clams. All LN clams died at 35 °C. LT_10_ (temperatures inducing 10% mortality) were 24, 28 and 36 °C in LN, CR and CWD clams, respectively.

### Molecular chaperones

**Figure 2:**
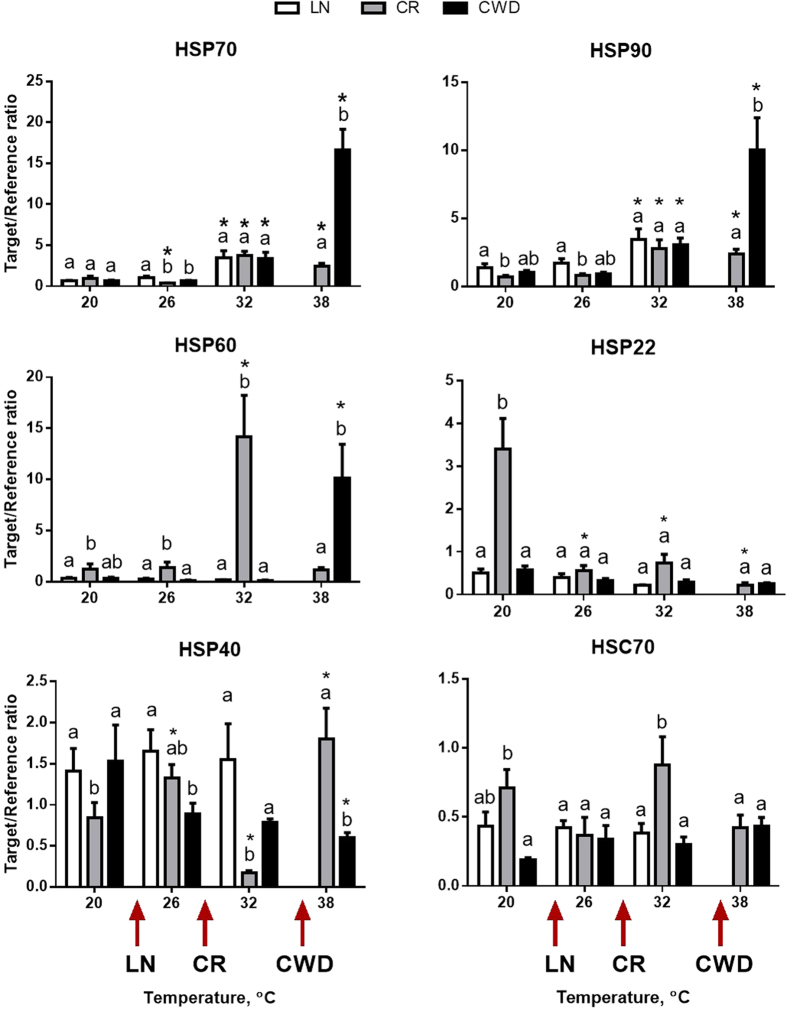
Effect of acute warming on expression of mRNA encoding molecular chaperones (heat shock proteins) in *C. fluminea* from the three studied populations. X- axis – exposure temperatures, Y- axis – mRNA levels of the target gene relative to the geometric mean of two reference genes (β-actin and RSP9). Different letters indicate significant differences between the means for different populations (p < 0.05). Asterisks indicate values that are significantly different from the respective control group (exposed at 20 °C) within each population (p < 0.05). Arrows on the X-axis indicate LT_10_ for each of the studied populations. Vertical bars represent the standard error of means. N = 8–9.

Levels of mRNA encoding molecular chaperones were significantly affected by the site, the experimental temperature and their interaction, except for HSC70 where only the site effect was statistically significant (Table 1). Elevated temperatures (32 °C) led to a significant upregulation of mRNA for the inducible HSP70 (by ~4–5 fold) and HSP90 (by ~2.5–4 fold) in all three studied populations (Fig. 2). Notably, mRNA expression of HSP70 and HSP90 increased slightly at 38 °C in CR clams, but rose dramatically (by ~25 and ~10 fold for HSP70 and HSP90, respectively) in CWD clams (Fig. 2). No data for 38 °C are available for LN clams due to 100% mortality. Expression of the constitutive HSP70 isoform (HSC70) was unaffected by the temperature in clams from the three studied populations (Fig. 2).

Mitochondrial HSP60 was strongly upregulated at 32 °C in the CR population (by ~11 fold) and at 38 °C in the CWD population (by ~30 fold), but did not change with exposure temperature in the LN population (Fig. 2). Expression of HSP60 mRNA expression decreased back to the baseline levels at the extreme temperature (38 °C) in CR clams.

Expression of mRNA for small HSPs (HSP40 and HSP22) did not change in response to acute temperature rise in LN clams (Fig. 2). In CR clams, expression of HSP22 mRNA declined at elevated temperatures, whereas HSP40 expression transiently declined at 32 °C and then increased at 38 °C. In CWD clams, HSP22 expression was not affected by the experimental temperature, while HSP40 levels decreased with warming (Fig. 2).

### Antioxidants and glutathione system

**Figure 3:**
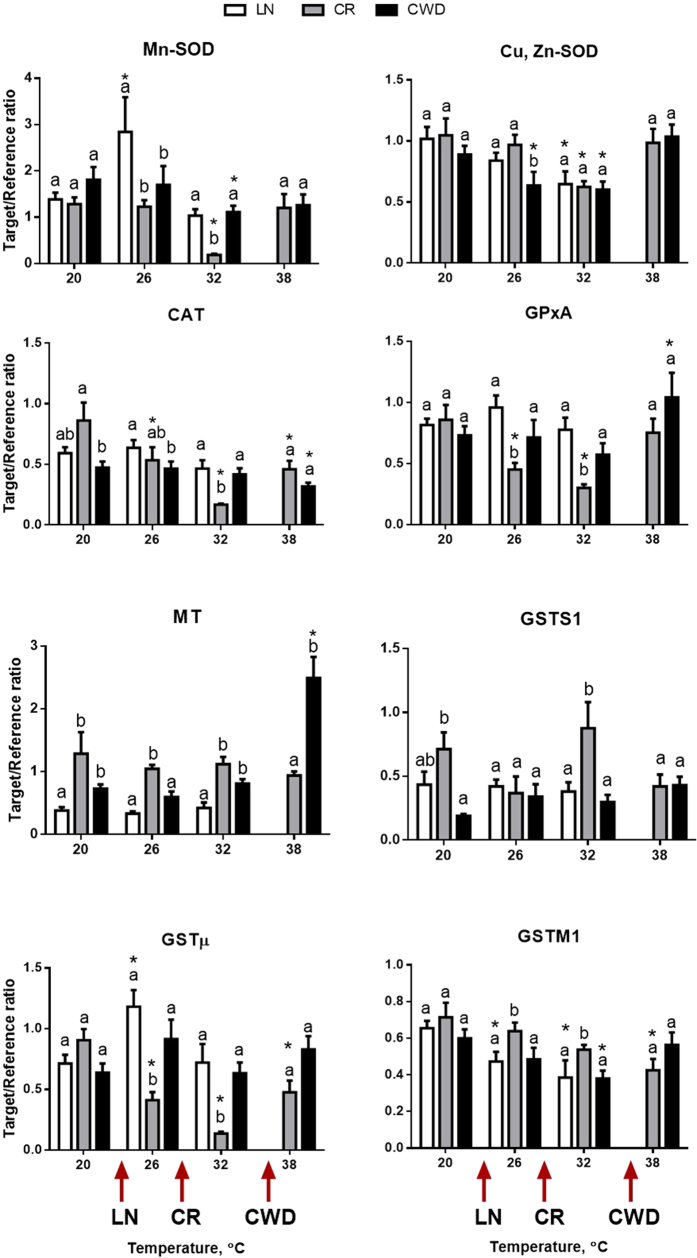
Effect of acute warming on expression of mRNA encoding antioxidants in *C. fluminea* from the three studied populations. X- axis – exposure temperatures, Y- axis – mRNA levels of the target gene relative to the geometric mean of two reference genes (β-actin and RSP9). Different letters indicate significant differences between the means for different populations (p < 0.05). Asterisks indicate values that are significantly different from the respective control group (exposed at 20 °C) within each population. Arrows on the X-axis indicate LT_10_ for each of the studied populations. Vertical bars represent the standard error of means. N = 8–9.

mRNA expression of all studied antioxidants (except GSTS1) was significantly affected by the temperature and/or site × temperature interactions (Table 1). Temperature-induced changes in the mRNA expression of antioxidants differed between the clams from the three study sites. In the LN population, exposure to 26 °C led to a significant upregulation of the expression of Mn-SOD, GSTμ and TXNRD1; this increase was transient and mRNA expression of these genes returned to the background levels at higher temperatures (32 and 38 °C) (Fig. 3). mRNA expression of CAT, GPxA, GSTS1, TPX and TXNIP did not change in response to acute temperature rise in LN clams, while expression of Cu, Zn-SOD and GSTM1 declined at 32 °C. The CR population showed the strongest response of the antioxidant expression to experimental heating. In this group, many antioxidants (Mn-SOD, Cu,Zn-SOD, CAT, GPxA, GSTμ and TXRND1) showed a similar non-linear expression pattern with a decrease in the mid-range of the experimental temperatures (26 and 32 °C) followed by the return to the baseline levels at 38 °C (Fig. 3). In the most heat-tolerant CWD population, expression levels of antioxidants generally remained stable during the acute temperature rise except for GPxA, MTs and TPX1 that were upregulated and CAT that was downregulated at the extreme temperature (38 °C).

### Oxidative lesions

**Figure 4:**
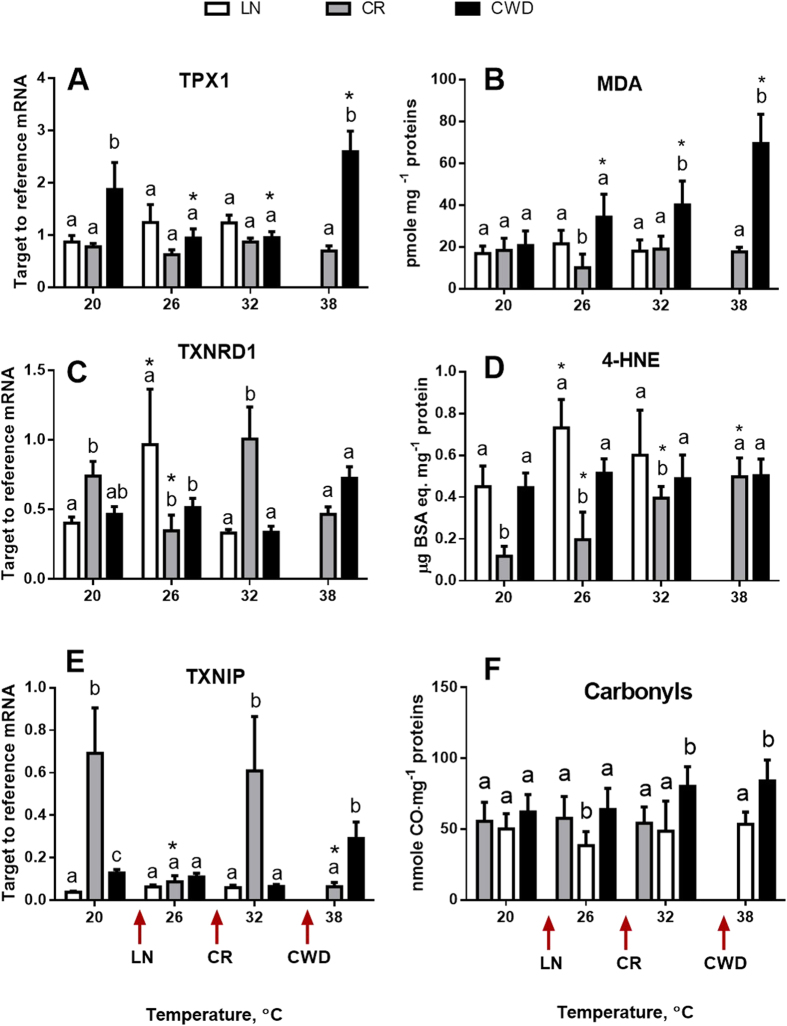
Effect of acute warming on expression of mRNA encoding components of thioredoxin system (**A,C,E**) and oxidative lesions (**B,D,F**) in *C. fluminea* from the three studied populations. X- axis – exposure temperatures, Y axis – mRNA levels of the target gene relative to the geometric mean of two reference genes (β-actin and RSP9) (**A,C,E**) or tissue levels of oxidative lesions (**B,D,F**). Different letters indicate significant differences between the means for different populations (p < 0.05). Asterisks indicate values that are significantly different from the respective control group (exposed at 20 °C) within each population (p < 0.05). Arrows on the X-axis indicate LT_10_ for each of the studied populations. Vertical bars represent the standard error of means. N = 8–9 for mRNA expression and 5 for oxidative lesions (MDA, 4-HNE, and carbonyls).

The levels of MDA and protein carbonyls were significantly affected by the site, but not experimental temperature, while the levels of 4-HNE were affected by the site, experimental temperature and their interaction (Table 1). Under the control conditions, the levels of MDA and carbonyls were similar in clams from all three studied populations, whereas the levels of 4-NHE were lower in the CR group (Fig. 4). Acute warming led to an increase of MDA levels in the CWD population and an increase of 4-HNE levels in the CR population from 26 °C onwards. Levels of 4-HNE were also slightly elevated at 26 and 32 °C in the LN populations, but this trend was only significant at 26 °C (Fig. 4). Elevated temperature had no effect on the protein carbonyl levels in any of the three studied populations (Fig. 4).

### Protein ubiquitination

**Figure 5:**
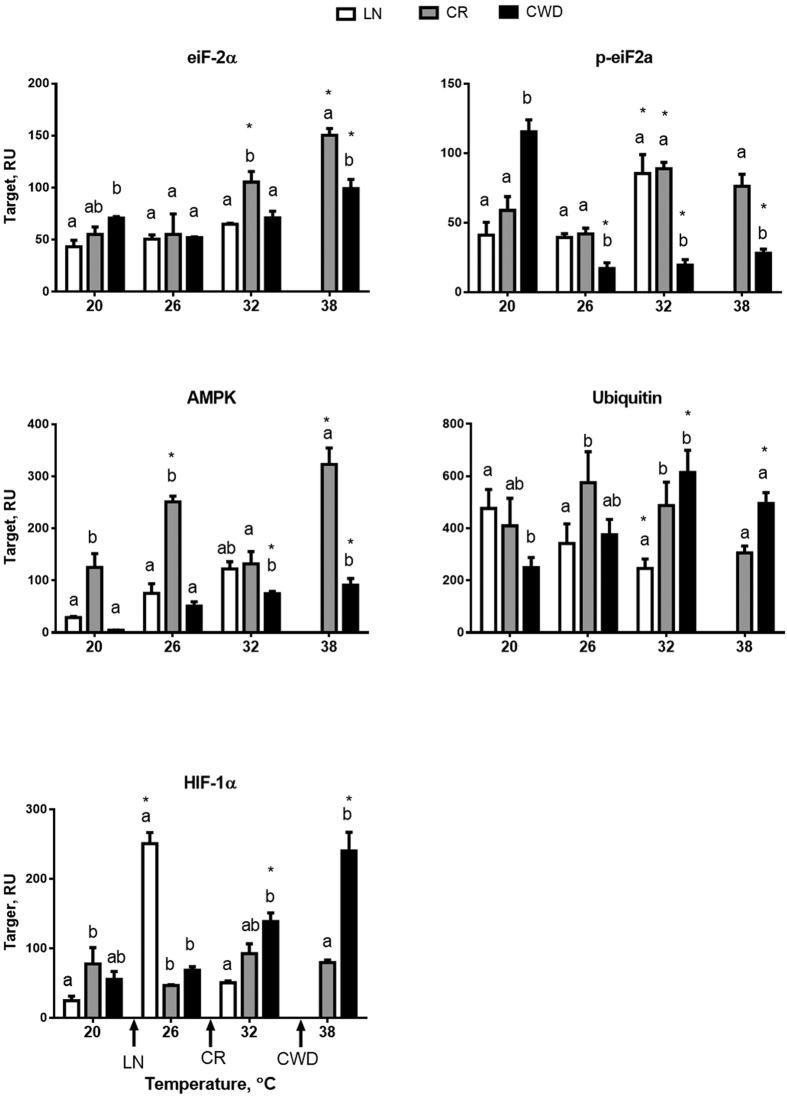
Effect of acute warming on protein expression of bioenergetic and protein synthesis biomarkers in *C. fluminea* from the three studied populations. X- axis – exposure temperatures, Y - axis – target protein levels in relative units (RU). Different letters indicate significant differences between the means for different populations (p < 0.05). Asterisks indicate values that are significantly different from the respective control group (exposed at 20 °C) within each population (p < 0.05). Arrows on the X-axis indicate LT_10_ for each of the studied populations. Vertical bars represent the standard error of means. N = 3–4 for all proteins except ubiquitin (N = 5) and HIF-1α from the heat exposed CR groups (26, 32 and 38 °C) where N = 2.

The baseline levels of ubiquitinated proteins were lower in CWD clams compared to those from the LN and CR sites (Fig. 5). The levels of protein ubiquitination increased with the increasing temperature in CWD clams, did not change in CR clams and decreased with temperature in LN clams (Fig. 5).

### Protein translation

Protein expression levels of the total and phosphorylated elongation factor 2α (eIF-2α and p-eIF-2α, respectively) were affected by the site, experimental temperature and their interaction (Table 1). Under the control conditions, protein levels of eIF-2α and p-eIF-2α were higher in the mollusks from the CWD group. Total levels of eIF-2α increased with increasing temperatures in CR and CWD but not LN clams (Fig. 5). Elevated temperature led to a strong decrease (by 4–7 fold) of the phosphorylated p-eIF-2α in the CWD clams and an increase of p-eIF-2α levels in the LN and CR groups (Fig. 5).

### Bioenergetic markers

Basal AMPKα expression levels were highest in the CR population and lowest in CWD clams. In all three populations, AMPK levels increased with increasing temperatures (Fig. 5). Expression of HIF-1α was induced by elevated temperature in the LN and CWD populations (at 26 and 32–38 °C, respectively) (Fig. 5). No temperature-induced change in the HIF-1α protein levels was found in CR clams.

**Figure 6:**
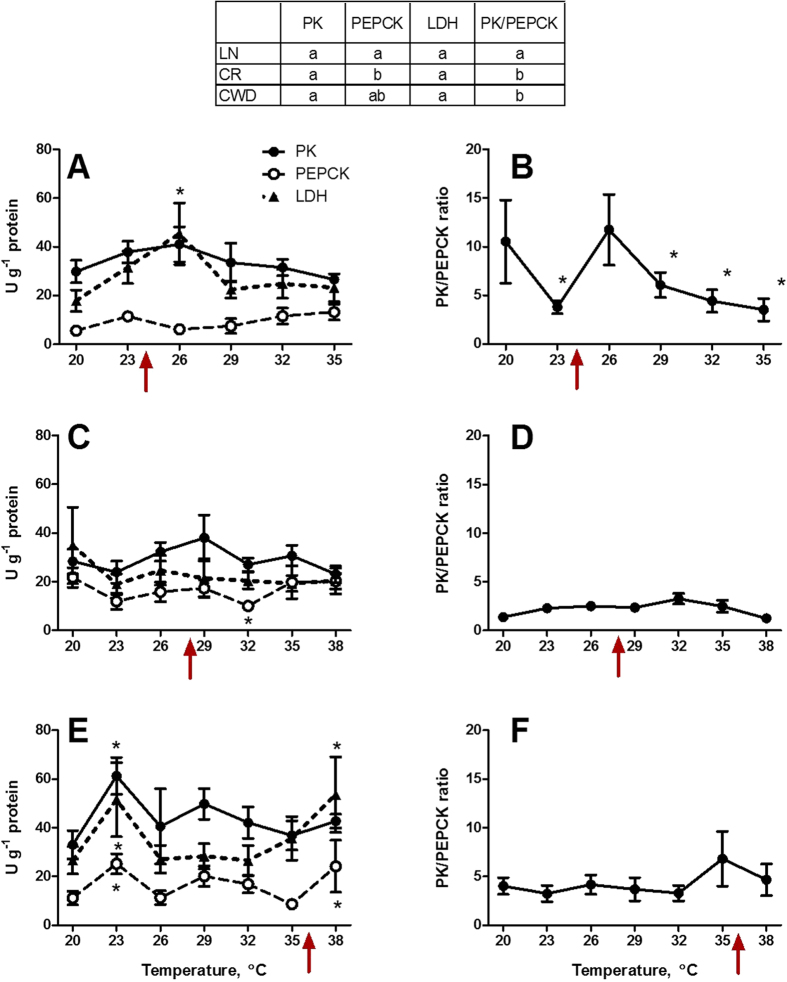
Effect of acute warming on enzyme activities in *C. fluminea* from the three studied populations. (**A,B**) – population LN, (**C,D**) – population CR, and (**E,F**) – population CWD. A, C, D – activities of PK, PEPCK and LDH; B, D, F – PK/PEPCK ratio. X- axis – exposure temperatures, Y - axis – specific activities of the respective enzymes (U g^−1^ protein). Asterisks indicate values that are significantly different from the respective control group (exposed at 20 °C) within each population (p < 0.05). Arrows on the X-axis indicate LT_10_ for each of the studied populations. An inset table above the graphs indicates comparisons of the enzyme activities between the control groups from different populations; if the rows do not share a letter, the respective enzyme activities are significantly different (p < 0.05). Vertical bars represent the standard error of means. N = 8.

Enzymatic activities of PK and LDH were similar in the control clams from the three studied populations, while the PEPCK activity was lower in LN clams compared to those from the CR and CWD sites (Fig. 6). As a result, PK/PEPCK activity (an index of the relative aerobic capacity) was considerably higher in the LN population compared to CR and CWD. Acute warming had little effect on the activities of PK, PEPCK and LDH (Fig. 6). In LN clams, acute warming led to a significant decrease in PK/PEPCK ratio indicating a decline in aerobic capacity and activation of anaerobic pathways (Fig. 6). In CR and CWD clams, PK/PEPCK ratio remained at the steady-state levels throughout the experimental exposures (Fig. 6).

### Data integration

Expression levels of different antioxidants (including Mn-SOD, Cu, ZN-SOD, GPxA, TPX, TXNRD1, CAT, GSTM1 and GSTμ) significantly and positively correlated with each other in *C. fluminea* across the studied populations and experimental groups (Supplementary Table 1). Tissue levels of oxidative lesions (MDA, 4-HNE and protein carbonyls) positively correlated with each other and negatively correlated with mRNA expression of select antioxidants (CAT in the case of MDA, MTs and GSTM1 in the case of 4-HNE, and GSTM1 and GSTS1 in the case of carbonyls). Notably, expression of HIF-1α positively correlated with the expression of molecular chaperones (HSP70, HSP90, and HSP22), antioxidants (MT, Mn-SOD, GSTM1, GSTμ, GPxA, TPX, and TXNRD1), oxidative lesions (MDA, HNE and carbonyls) and activity of LDH. Expression of total eiF-2α was positively correlated with the expression of its phosphorylated form (p-eiF-2α), as well as AMPK, MTs and molecular chaperones (HSP60, HSP70, HSP90 and HSP22) and negatively correlated with the expression of antioxidants (Mn-SOD, GSTM1, GSTμ, and CAT) (Supplementary Table 1). Expression of p-eiF-2α also negatively correlated with oxidative stress parameters (GSTμ, MDA and carbonyls) and ubiquitin levels (Supplementary Table 1). AMPK expression positively correlated with the activity of PEPCK, and negatively - with the PK activity, expression of antioxidants (GSTμ, TPX1) and oxidative lesions (MDA, HNE and carbonyls) (Supplementary Table 1).

**Figure 7:**
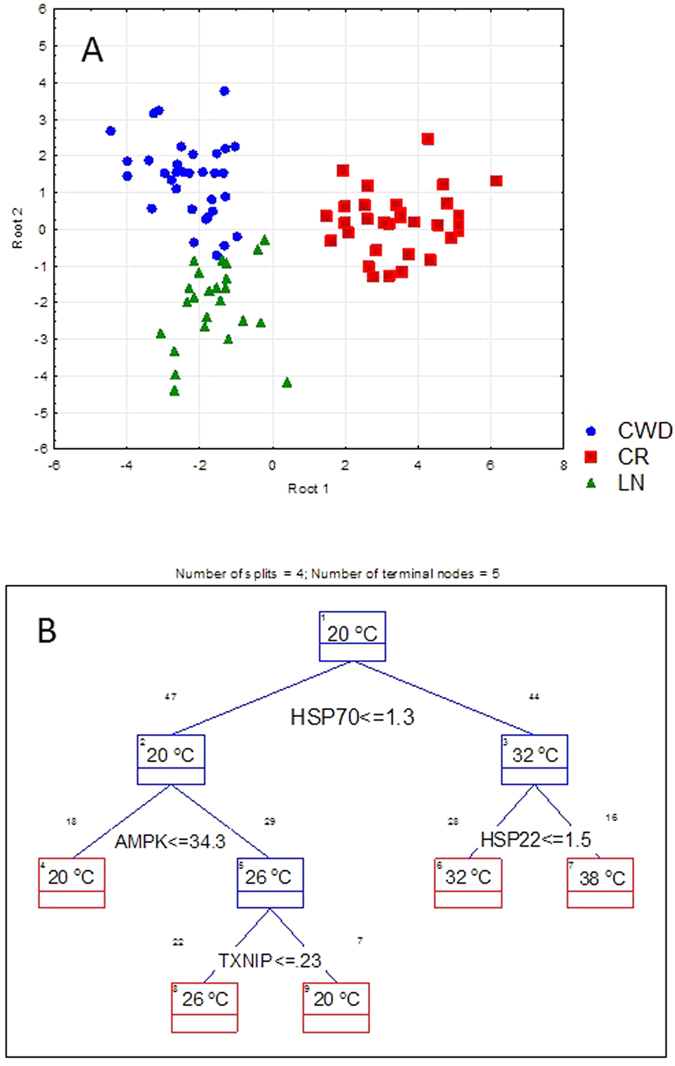
Separation of the studied populations and/or experimental treatment groups based on the discriminant (**A**) and CART (**B**) analyses. A - Population groupings based on the discriminant analysis. Biomarkers that significantly contributed to the discriminant function included Mn-SOD, HSP22, MDA, 4-HNE, AMPK and eiF-2α (p < 0.05). B – Classification and regression Tree (CART) analysis. The main distinguishing characteristic separating each of the temperature groups (across all populations) is shown at the respective nodes of the tree.

Discriminant analysis showed a clear separation among the three studied populations based on their site of origin (F_48,130_ = 10.59, p < 0.0000) and the experimental temperature (F_72,192_ = 10.45, p < 0.0000). The parameters that most significantly contributed to the discriminant function separating different populations included Mn-SOD, HSP22, MDA, 4-HNE, eiF-2α, and AMPK (Fig. 7A). CART analysis (used to identify a single most distinguishing characteristic between the groups) showed that clams exposed to 32 or 38 °C were distinguished from other experimental temperatures by high levels of HSP70, while clams exposed to 38 °C were differentiated by higher HSP22 expression levels from those exposed to 32 °C (Fig. 7B). Clams exposed to 26 °C were distinguished from the control group (20 °C) based on the expression of AMPK and TXNIP.

## Discussion

Long-term acclimation of *C. fluminea* to a chronically warmed CWD area was associated with a dramatic increase in heat tolerance compared to the clams from the habitats that do not experience thermal pollution. In the CWD population, ~90% clams could withstand 48 h of exposure at 38 °C, the conditions which where lethal to >90% of the CR clams and 100% of the LN clams. Furthermore, LT_10_ (i.e. the threshold temperature at which 10% of the population died) was 12 °C higher in the CWD clams than in the LN clams, and 8 °C higher compared to the CR clams. While we cannot rule out the possibility that other ecological variables (such as food availability in the field) may have contributed to the variation in thermal tolerance, a strong correlation between the thermal tolerance limits of clams and daytime summer temperatures in their habitats make the thermal regime the most plausible explanation for the observed differences in the thermal thresholds. This plasticity of the upper thermal limits indicates a strong potential of *C. fluminea* to withstand long-term warming such as expected during GCC. It is worth noting that the upper thermal limit of the most tolerant CWD population is close to the maximum thermal tolerance limits recorded in ectotherms and thus may have a limited capacity to evolve to reach even higher levels^20^.

A common pattern of heat stress response in all studied populations included upregulation of mRNA for large mitochondrial and cytosolic heat shock proteins (HSP70 and HSP90). Induction of HSP70 and HSP90 is a common response to heat stress in many organisms including mollusks^21^,^22^,^23^,^24^,^25^, reflecting the important role of these chaperones in refolding the partially denatured proteins and targeting the irreparably damaged ones for degradation^26^,^27^. Temperature-dependent chaperone expression revealed significant interpopulational differences linked to the differences in the heat tolerance among the three studied populations. While the magnitude of HSP70 and HSP90 induction at intermediate temperatures (32 °C) were similar in the three studied populations, only the heat tolerant CWD clams were capable of a strong upregulation (by 10–25 fold) of HSP70 and HSP90 at the extreme temperature (38 °C). Given the protective role of HSP70 and HSP90 against the heat-induced protein damage^26^,^28^, high levels of HSP70 and HSP90 expression in the CWD clams may contribute to their ability to survive extremely high temperatures. In marine gastropods from the genus *Tegula*, acclimation to elevated temperatures led to an upward shift of the temperature, at which the highest HSP expression was observed^22^. Interestingly, the temperature at which the synthesis of HSPs was inactivated strongly correlated with the species-specific thermal tolerance^22^. In sea urchins (*Strongylocentrotus purpuratus*) and fish (*Gillichthys mirabilis*) long-term acclimation to elevated temperatures resulted in better survival and enhanced transcription of HSP70 at extreme temperatures (32–36 °C) compared to their cold-acclimated counterparts that failed to induce HSP70 mRNA under these conditions^29^,^30^. A strong link between the ability to synthesize HSP70 at extreme temperatures and thermotolerance was also found in ascidians, where the more thermotolerant invasive species *Diplosoma listerianum* expressed high levels of HSP70 at extreme temperatures while the less thermotolerant, native *Distaplia occidentalis* failed to maintain HSP70 levels at the temperatures above LT_50_^31^. In two species of freshwater unionid bivalves, a more thermotolerant species*, Villosa lienosa*, expressed higher levels of HSP70 and HSP90 transcripts during heat stress compared to the thermally sensitive *Villosa nebulosa*^32^. These data indicate that an ability to maintain synthesis of HSP70 and HSP90 at extreme temperatures plays an important role in setting the upper limit of thermal tolerance in aquatic invertebrates, including mollusks.

High heat tolerance of CWD clams was also associated with the ability to maintain high levels of the protein synthesis during acute heat rise as indicated by an increase in the levels of the eukaryotic transcription factor eiF2α and a decrease of its phosphorylated form. In mollusks like in other eukaryotes, eiF2α is an essential translation initiation factor required for protein translation which mediates the binding of tRNA for methionine to the ribosome^33^,^34^. eiF2α can be inactivated by reversible phosphorylation which results in suppression of the protein synthesis^33^,^34^. The temperature-induced increase in the amount of active, non-phosphorylated eiF2α in CWD clams indicates high translational activity at elevated temperatures. This will support the *de novo* synthesis of heat shock proteins (such as HSP70, HSP90 and HSP60), which are essential for survival under the conditions of heat stress^26^,^27^. In contrast, in the two less heat-tolerant populations (LN and CR) the levels of phosphorylated eiF2α increased with increasing temperatures indicating suppression of the protein translation. Given that HSPs are the group of the proteins predominantly synthesized during heat stress in mollusks^21^,^22^,^35^, suppression of the protein synthesis rates at elevated temperatures may impair heat shock response leading to protein damage and mortality in heat-sensitive clam populations.

Notably, the temperature-dependent mRNA expression of HSP40, a co-chaperone of HSP70, did not correlate with the HSP70 expression profile in *C. fluminea*. HSP40 regulates HSP70 function and acts in concert with HSP70 to prevent protein aggregation and catalyze re-folding of misfolded proteins^36^,^37^. Given the tight functional linkages between HSP40 and HSP70^36^,^37^, one might expect coordinated changes in mRNA expression of these two chaperones during acute temperature rise. However, mRNA levels of HSP40 remained stable during the acute temperature rise in LN clams and decreased at elevated temperatures in CR and CWD clams. This indicates that transcriptional regulation of HSP70 and HSP40 is decoupled, and that unlike HSP70, mRNA expression of HSP40 is not a good indicator of heat stress in *C. fluminea*.

Transcript levels of another small molecular chaperone, HSP22, were temperature-insensitive in LN and CWD clams and suppressed during the temperature rise in CR clams. In marine intertidal mussels *Mytilus californianus* expression of a small HSP (HSP30) decreased at elevated temperatures^38^ similar to the pattern seen in the CR population. To date, the functional roles and temperature-dependent regulation of small heat shock proteins (sHSPs) such as HSP22 and HSP30 are not well understood in mollusks. In mammals, sHSPs are involved in regulation of apoptosis, antioxidant defense and copper homeostasis^39^,^40^. In a model invertebrate (*Drosophila melanogaster*) HSP22 localizes to the mitochondria and has been proposed to play a role in protection of the mitochondria from oxidative stress^41^. Interestingly, heat-induced suppression of HSP22 in CR clams goes hand-in-hand with a decrease in the transcript levels of mitochondrial and cytosolic antioxidants such as Mn-SOD, Cu, Zn-SOD, CAT, GPX and GSTμ. This raises an intriguing possibility that HSP22 may have an antioxidant function in mollusks similar to that in *Drosophila* and mammals^40^,^41^, which requires further investigation. Regardless of the cellular functions of HSP22, mRNA levels of this gene do not show a consistent pattern of change during temperature rise in different clam populations and thus cannot serve as a biomarker for heat stress in *C. fluminea*.

The highly and intermediately tolerant populations (CR and CWD) strongly overexpressed the mitochondrial chaperone HSP60 at 32 and 38 °C, respectively, whereas no temperature-dependent induction of HSP60 mRNA was observed in heat-sensitive LN clams. Mitochondrial HSP60 is involved in maintaining the mitochondrial function and DNA stability^42^ and can protect mitochondria against temperature stress^42^,^43^,^44^ as well as other proteotoxic insults such as exposure to toxic metals^23^,^45^,^46^. Inability of LN clams to overexpress HSP60 during acute heat stress may contribute to the damage of their mitochondria and lead to a decrease in the aerobic capacity at elevated temperatures. This hypothesis is supported by a decrease in the PK/PEPCK ratio in LN clams at elevated temperatures (≥29 °C). In molluscs, PK and PEPCK act as a metabolic switch channeling glycolytic substrates to aerobic (PK) or anaerobic (PEPCK) ATP production in mitochondria^16^,^47^. Thus, a decrease in PK/PEPCK ratio reflects a decrease in the aerobic capacity for ATP production and activation of anaerobic pathways^16^,^18^,^47^. In contrast to LN clams, CR and CWD clams maintained the steady-state PK/PEPCK ratio at all studied temperatures. This suggests that the mitochondrial metabolism of CR and CWD clams is robust to the acute temperature rise, possibly due to the protective overexpression of the mitochondrial chaperone HSP60 at high temperatures.

Tissue levels of AMPKα increased with increasing temperatures in clams from all three populations of *C. fluminea*, albeit the trend was only significant in CR and CWD clams. This may indicate cellular energy deficiency at elevated temperatures, since AMPKα is the main cellular metabolic sensor activated in response to the energy deficit^48^. Earlier studies in marine mollusks and crabs also showed upregulation of AMPK during heat stress resulting in activation of ATP-generating pathways to support energy homeostasis^49^,^50^. This hypothesis is also consistent with accumulation of HIF-1α protein in clams at elevated temperatures indicative of tissue hypoxemia. HIF-1α is a constitutively expressed transcription factor which is rapidly degraded in the presence of oxygen but accumulates during hypoxia and/or tissue hypoxemia^15^,^51^ triggering an adaptive transcriptional program that enhances oxygen delivery to the tissue, increases glucose uptake, and stimulates glycolysis^51^,^52^. Upregulation of AMPKα and stabilization of HIF-1α in *C. fluminea* tissues indicates heat-induced impairment of energy status and potential hypoxemia. However, this cellular energy stress was not sufficient to induce transition to partial anaerobiosis as shown by the stability of PK/PEPCK ratio at the temperatures that trigger accumulation of AMPKα and HIF-1α in the studied clam populations. Oxidative stress response was activated at the intermediate temperatures (26 °C) in the heat-sensitive LN clams as indicated by the elevated mRNA expression of Mn-SOD, GSTμ and TXNRD1. In the intermediately and highly heat-tolerant CR and CWD clams mRNA expression for antioxidants remained at the background levels or declined at elevated temperatures (with the exception of TPX1 and GPxA mRNA upregulated in the CWD clams at 38 °C). This indicates that transcriptional upregulation of antioxidants does not play a key role in the mechanism of heat tolerance in *C. fluminea*. Lack of induction and/or suppression of mRNA levels of antioxidants at the elevated temperatures (such that induce molecular chaperones) may indicate antagonistic interactions between the transcriptional activation of antioxidant response and heat shock pathways in *C. fluminea*. Similar antagonism between the heat shock (HSR) and oxidative stress (OxSR) response has been shown in a model organism *C. elegans*^53^. Prioritization of heat shock over antioxidant response may come at a cost of oxidative damage to the tissues, as indicated by accumulation of oxidative lesions (MDA and 4-HNE) and ubiquitinated proteins in the CR and CWD clams at high temperatures (32–38 °C).

Despite the overall positive correlations between the expression levels of different antioxidants, no single antioxidant-encoding gene showed a consistent pattern of change in response to acute warming across all studied populations. This indicates that while acute heat stress affects redox status of *C. fluminea*, the compensatory response may involve different antioxidants. Oxidative stress is a common response to elevated temperature in aquatic ectotherms including bivalves^54^,^55^,^56^,^57^,^58^. However, similar to our present study, earlier studies of temperature-induced OxSR showed that specific players (i.e. genes and proteins involved in the OxSR) and the direction of change (i.e. upregulation or suppression) vary among species, habitats and exposure conditions^59^,^60^. This may reflect the molecular complexity and redundancy of the antioxidant protection systems in the cell, as well as the dual role of ROS as important signaling molecules, on one hand, and potentially damaging agents, on the other^61^. Our findings emphasize that detection of the oxidative stress signature during acute warming requires assessment of a broad range of antioxidants and redox status parameters in aquatic organisms (such as conducted in the present study).

As a corollary, exposure to the chronic, long-term warming in the CWD area strongly affects thermal tolerance of freshwater clams *C. fluminea* making them capable of surviving extremely high temperatures (up to 38 °C). High heat tolerance in clams from the CWD area is associated with their ability to maintain high aerobic capacity and active protein synthesis at elevated temperatures, and strong induction of cytosolic and mitochondrial molecular chaperones (including HSP70, HSP90 and HSP60) during heat stress. Notably, there appears to be a trade-off between the heat shock response and oxidative stress response in *C. fluminea*. At extreme temperatures, heat shock response is prioritized over the antioxidant protection, so that oxidative lesions accumulate in clam tissues. An increase in thermal tolerance and the associated molecular traits in the CWD clams (reflecting phenotypic plasticity, genetic adaptation or both) indicate that *C. fluminea* has a potential to survive global climate change in much of its current distribution range. Future studies are needed to determine whether such physiological flexibility may also increase the invasive spread of *C. fluminea* and help them outcompete less heat-tolerant indigenous freshwater bivalves as the mean temperatures and thermal fluctiuations (and thus frequencies of the thermal extremes) increase in freshwater habitats.

## Materials and Methods

### Animal collection and maintenance

Adult *C. fluminea* (shell length 26.2 ± 0.3 mm, N = 210) were collected in August 2015 from three habitats in North Carolina: 1) Lake Norman State Park (LN; 35 40′18.01 N, 80 55′57.02 W), 2) Catawba River (CR; 35 16′20.65 N, 81 00′41.25 W), and 3) the water discharge channel of the Catawba Nuclear Station (CWD; 35 02′42.42 N, 81 04′10.12 W). All three collection sites are situated in the Catawba River basin and characterized by sandy-clay bottom sediments, lack of underwater macrophyte vegetation and shallow depth (<2 m). The study sites are ~70 km apart with LN being the most upstream, CR – intermediate, and CWD – the most downstream location in the Catawba River basin (Fig. 1B). While the distance between the study locations makes exchange of the larvae unlikely, we cannot exclude the potential larval transport from the upstream to downstream sites. Therefore, the differences in thermal tolerance and molecular responses to the heat stress between clams from the three study sites may reflect genetic adaptation, phenotypic plasticity or both. Physico-chemical parameters of the water (pH, phosphates, nitrites, ammonium, sulfate, chloride, oxidisability, calcium hardness, and iron levels) collected at the three study sites were determined using Multitest Ammonia and Multitest Nitrite and Nitrate (Seachem Laboratories Inc., Madison, GA) for ammonia, nitrate and nitrite or the standard tests recommended by the American Public Health Association (APHA) for all other parameters^62^. All measured parameters were below the threshold levels established for the protection of freshwater aquatic life (EC Council Directive 98/83/EC; http://www.emwis.org/IFP/law_EU.htm) indicating that all study sites had good water quality and negligible levels of contamination (data not shown). Temperature was measured every other week in summer 2015 (July-September) and in spring 2016 (March-April) near the clam collection sites. In summer, water temperatures at the CWD site were ~8 °C higher than at the LN site and ~3 °C higher than at the CR site (29.7 ± 0.7 °C, N = 3; 33.8 ± 0.4 °C, N = 6; 37.5 ± 0.4 °C, N = 6 for LN, CR and CWD sites, respectively). In spring, water temperatures were similar at the CWD and CR sites (18.2 ± 0.8 °C, N = 4 at each site) and ~3 °C higher than at the LN site (15.8 ± 1.3 °C, N = 4).

Clams were acclimated for 7–10 days in the laboratory in recirculating aquaria (at the density of 50 clams per 30 l) containing dechlorinated, conditioned tap water at 20 °C, with acid-washed sand as a substrate. The conditioned tap water was used to ensure uniform high water quality in experimental treatments. Three replicate aquaria were used for each population. Clams were fed *ad libitum* using 2 ml of commercial algal blend (DT’s Premium Plankton Blend, DT’s Plankton Farm, Sycamore, IL) per tank every other day. After pre-acclimation, clams were exposed to an acute temperature increase from 20 °C to 41 °C, with the warming rate of 1 °C per hour followed by a 48 h acclimation period after every 3 °C rise. This warming reduces the acute metabolic effects of rapid temperature change but does not allow for full thermal acclimation^63^,^64^. Water pH, nitrite, nitrate and ammonia concentrations were measured daily. Ammonia and nitrite levels were <0.01 mg l^−1^, nitrate levels <2 mg l^−1^ (Multitest Ammonia and Multitest Nitrite and Nitrate, Seachem Laboratories Inc., Madison, GA), and pH was 7.13 ± 0.02 (N = 14) throughout the exposures. Mortality was recorded, and tissue samples were collected after 48 h exposure at each experimental temperature to assess stress biomarkers and activities of key metabolic enzymes. Due to the limited amount of tissues, different biomarkers were measured in different tissues of clams (Table 2). Oxidative stress biomarkers and molecular chaperones were assessed in the gills, which is the main organ of the gas exchange and thus most subjected to oxidative stress. Markers related to protein turnover and oxidative protein damage (carbonyl and MDA- and HNE-protein conjugates) were measured in the foot muscle. Enzyme activities were measured in the digestive gland, a key organ involved in energy storage and metabolism of bivalves.

### Immunoblotting

Foot muscle tissues were homogenized in ice-cold homogenization buffer (100 mM Tris, pH 7.4, 100 mM NaCl, 1 mM EDTA, 1 mM EGTA, 1% Triton-X 100, 10% glycerol, 0.1% sodium dodecylsulfate, 0.5% deoxycholate, 0.5 μg mL^−1^ leupeptin, 0.7 μg ml^−1^ pepstatin, 40 μg ml^−1^ phenylmethylsulfonyl fluoride and 0.5 μg ml^−1^ aprotinin), sonicated three times for 10 s each (output 69 W, Sonicator 3000, Misonix, Farmingdale, NY, USA), with cooling on ice between sonications, and centrifuged for 10 min at 14,000 × *g* and 4 °C. Protein content was measured in the supernatant using Bio-Rad Protein Assay kit (Bio-Rad Laboratories, Hercules, CA, USA), and protein expression was determined using standard immunoblotting techniques. Ubiquitinated proteins were analyzed by dot-blot technique using 96-well Convertible® dot/slot blotter. Protein samples (20 μg per well) were loaded onto pre-wet nitrocellulose membrane under vacuum, and probed with a polyclonal rabbit antibody against ubiquitin (#Z0458, Dako Denmark A/S, Grostrup, Denmark). All other proteins, including total and phosphorylated eukaryotic translation initiation factor (eiF-2α and p-eiF-2α, respectively), hypoxia-inducible factor 1α (HIF-1α), and 5′-AMP-activated protein kinase α (AMPKα) were resolved using SDS-PAGE electrophoresis. Protein samples (50 μg per lane for eiF-2α, p-eiF-2α, or 80 μg for AMPK and HIF-1α) were loaded onto 10% polyacrylamide gels and run at 100 mA for 2–2.5 h at room temperature. The resolved proteins were transferred onto a nitrocellulose (for AMPK and HIF-1α) or PVDF (for eiF-2α and p-eiF-2α) membrane in 96 mM glycine, 12 mM Tris and 20% methanol (v/v) using a Trans-Blot semi-dry cell (Thermo Fisher Scientific, Portsmouth, NH, USA). Equal loading was verified with Amido Black staining (McDonough *et al*. 2015). The membranes were blocked in 5% non-fat milk in Tris-buffered saline, pH 7.6, and probed overnight with primary monoclonal antibodies against p-EIF-2α (Ser51) (#07–760, Millipore, Temecula, CA, USA), EIF-2α (#AHO1182, Invitrogen, Frederick, MO, USA), AMPKα (#2793 S, Cell Signaling Technology, Danvers, MA, USA), and HIF-1α (#QC49321–42215, Avivasysbio, USA). After washing off the primary antibody, membranes were probed with the respective polyclonal secondary antibodies conjugated with horseradish peroxidase (Jackson Immunoresearch, West Grove, PA, USA) and proteins detected by enhanced chemiluminescence according to the manufacturer’s instructions (Pierce, Rockford, IL, USA). Densitometric analysis of the signal on SDS-PAGE membranes and dot-blots was performed by GelDoc 2000™ System with Quantity One 1D Analysis Software (Bio-Rad Laboratories Inc., Hercules, CA, USA). To ensure that the heat stress-induced differences in the protein levels are not confounded with blot-to-blot variations, each blot included the same control sample as an internal standard. All antibodies produced bands of the expected molecular size as specified by the respective manufacturers (Supplementary Figure S1). All antibodies except HIF-1α produced a single band. Western blots for HIF-1α included two bands: an upper band (~110 kDa) corresponding to HIF-1α and a lower diffuse band of ~70 kDa corresponding to HIF-1α degradation products. Only the upper band corresponding to the intact transcription factor HIF-1α was quantified.

### Enzyme activities

Activities of PK (EC 2.7.1.40), PEPCK (EC 4.1.1.31) and LDH (EC 1.1.1.27) were measured at 20 °C in hepatopancreas of mollusks using standard NADH-coupled spectrophotometric assays as described elsewhere^8^,^18^. A molar extinction coefficient of 6.22·10^6^ M^-1^·cm^−1^ for NADH was used. In mollusks, PK channels glycolytic substrates to aerobic catabolism^47^,^65^. PEPCK and LDH were chosen as they are involved in anaerobic production of succinate and lactate, two major anaerobic end-products in freshwater mollusks^65^. For all enzyme activities, assay conditions were selected to ensure non-limiting concentrations of the substrates and linearity of the reactions over the assay time. The initial reaction velocity was used to estimate V_max_. Enzyme activities (V_max_) were expressed as U g^−1^ protein.

### Quantitative real-time PCR (qRT-PCR)

Total RNA was extracted from the gills tissue of mollusks with TRIzol® reagent (Invitrogen, Carlsbad, USA). Specific primers for target genes were designed against partial mRNA sequences published in the National Center for Biotechnology Information (NCBI) database (Table 1) using PrimerQuest software (Integrated DNA Technologies, Inc., Coralville, IA, USA). RNA amount and quality were assessed using Nanodrop spectrophotometer. RNA concentration in all samples was >200 ng/μL with A260/280 and A260 ⁄230 values of 2.0 ± 0.1 and 2.2 ± 0.1, respectively (N = 130). All RNA samples were subjected to a PCR reaction with gene-specific primers (Table 1) to test for DNA contamination; no amplification was observed after 40 cycles of PCR indicating that RNA samples contained no DNA contamination that could interfere with qRT-PCR. cDNA was then synthesized from RNA samples as described elsewhere^66^. Transcript levels of target genes as well as ß-actin and 40 S ribosomal protein S9 (RSP9) used as the reference genes were quantified by qRT-PCR using a 7500 Fast Real-Time PCR System (Life Technologies, Carlsbad, CA, USA) and SYBR® Green PCR kit (Life Technologies, Bedford, MA, USA). Expression levels of ß-actin and RSP9 did not significantly differ between the experimental groups (p > 0.05). The normalized expression of target genes was calculated as follows:


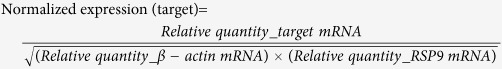


Relative mRNA quantities of the target and reference genes were calculated according to Pffafl (2001) using gene-specific amplification efficiencies. Amplification efficiency was determined in each run by amplifying serial dilutions of the internal cDNA standard (Pfaffl, 2001). The qRT-PCR conditions were as described in previous studies^58^,^66^; the annealing and read temperatures were 55 °C and 72 °C, respectively, for all primer pairs except HSC70. For HSC70 the annealing and read temperatures were 50 °C and 72 °C, respectively. A single cDNA sample was included in each run to test and correct for the potential run-to-run amplification variability^58^,^67^.

### Oxidative lesions

Protein conjugates of malondialdehyde (MDA) and 4-hydroxynonenal (4-HNE) were measured in gill tissue homogenates (N = 5) using enzyme immunoassays (MDA OxiSelect™ MDA adduct ELISA Kit and HNE OxiSelect™ HNE-His adduct ELISA Kit, respectively) according to the manufacturer’s protocols (Cell Biolabs, Inc., CA, USA). Protein carbonyl (PC) content was measured in gill tissue homogenates through the reaction with 2,4-Dinitrophenylhydrazine (DNPH)^68^. The differences in absorbance between the DNPH- and the HCl-treated samples were determined by spectrophotometry at 360 nm, and the amount of carbonyl was determined by using a molar extinction coefficient of 2.2·10^4^ M^−1^·cm^−1^. Data were expressed as nmol carbonyl·mg^−1^ of soluble extracted protein.

### Statistical analysis

Data were tested for normality and homogeneity of variance by Kolmogorov-Smirnoff and Levene’s tests, respectively. As needed, data were normalized by Box-Cox common transforming method (Table 1). Fisher’s Least Significant Differences (LSD) tests were used for planned post-hoc comparisons of the differences between the pairs of means of interest. Logit analysis was used to calculate LT_10_ and LT_50_, the temperatures causing 10% and 50% mortality, respectively. Pearson’s correlation was used to assess correlations between the studied traits within each individual across all experimental groups. Normalized Box-Cox transformed data were subjected to the discriminant analysis to reduce the dimensionality of the multivariate data set and determine the grouping of the studied treatment groups in the multivariate trait space. The classification tree based on all the studied traits was built using Classification and Regression Tree (CART) analysis (as implemented in Statistica v. 10.0) using raw (non-transformed) data. CART analysis creates a tree based on a set of decision rules that identify homogeneous groups as a function of a set of explanatory variables (e.g. the values of specific biomarkers) and permits identification of a single most distinguishing characteristic at each split of the classification tree^69^. All statistical analyses were performed with Statistica v. 10.0. Differences were considered significant if the probability of Type I error was less than 0.05. For all traits and all experimental treatment groups, sample size was 8–13 except of immunoblotting analysis where sample size was 5. The data are presented as means ± standard error (SE) unless indicated otherwise.

## Additional Information

**How to cite this article**: Falfushynska, H. I. *et al*. Long-Term Acclimation to Different Thermal Regimes Affects Molecular Responses to Heat Stress in A Freshwater *Clam Corbicula Fluminea. Sci. Rep.* **6**, 39476; doi: 10.1038/srep39476 (2016).

**Publisher's note:** Springer Nature remains neutral with regard to jurisdictional claims in published maps and institutional affiliations.

## Supplementary Material

Supplementary Information

## Figures and Tables

**Table 1:** ANOVA: Effects of the experimental temperatures and the site of origin and their interactions on the studied traits of *C. fluminea*.

Cellular process	Biomarker	Site	Temperature	Site × Temperature
	Gill tissue			
OS	Mn-SOD	**F**_**1,90**_** = 28.1 P < 0.001**	**F**_**2,90**_** = 36.2 P < 0.001**	**F**_**5,90**_** = 9.3 P < 0.001**
OS	Cu,Zn-SOD	F_1,90_ = 3.9 P = 0.05	**F**_**2,90**_** = 13.4 P < 0.001**	F_5,90_ = 1.5 P = 0.202
OS	CAT	F_1,90_ = 0.08 P = 0.772	**F**_**2,90**_** = 24.1 P < 0.001**	**F**_**5,90**_** = 9.3 P < 0.001**
OS	GSTμ	**F**_**1,90**_** = 41.2 P < 0.001**	**F**_**2,90**_** = 21.6 P < 0.001**	**F**_**5,90**_** = 12.9 P < 0.001**
OS	GSTM1	F_1,90_ = 2.9 P = 0.095	**F**_**2,90**_** = 13.1 P < 0.001**	**F**_**5,90**_** = 2.9 P = 0.018**
OS	GSTS1	**F**_**1,90**_** = 4.6 P = 0.035**	F_2,90_ = 0.09 P = 0.914	F_5,90_ = 2.3 P = 0.051
OS	GPxA	**F**_**1,90**_** = 9.8 P = 0.002**	**F**_**2,90**_** = 9.3 P < 0.001**	**F**_**5,90**_** = 3.0 P = 0.015**
OS	TPX1	**F**_**1,90**_** = 30.6 P < 0.001**	F_2,90_ = 1.3 P = 0.283	**F**_**5,90**_** = 4.8 P = 0.001**
OS	TXNLP	F_1,90_ = 1.0 P = 0.313	F_2,90_ = 2.0 P = 0.147	**F**_**5,90**_** = 9.8 P < 0.001**
OS	TXNRD1	F_1,90_ = 0.89 P = 0.348	F_2,90_ = 0.26 P = 0.774	**F**_**5,90**_** = 9.2 P < 0.001**
OS	MTs	F_1,90_ = 0.72 P = 0.400	F_2,90_ = 0.40 P = 0.670	**F**_**5,90**_** = 4.3 P = 0.002**
CH	HSP90	**F**_**1,90**_** = 16.8 P < 0.001**	**F**_**2,90**_** = 33.9 P < 0.001**	**F**_**5,90**_** = 3.4 P = 0.008**
CH	HSC70	**F**_**1,90**_** = 4.6 P = 0.035**	F_2,90_ = 0.09 P = 0.914	F_5,90_ = 2.3 P = 0.051
CH	HSP70*	**F**_**1,90**_** = 18.2 P < 0.001**	**F**_**2,90**_** = 100.9 P < 0.001**	**F**_**5,90**_** = 14.8 P < 0.001**
CH	HSP60*	**F**_**1,90**_** = 35.6 P < 0.001**	**F**_**2,90**_** = 5.7 P = 0.005**	**F**_**5,90**_** = 22.7 P < 0.001**
CH	HSP40	F_1,90_ = 2.2 P = 0.143	**F**_**2,90**_** = 11.4 P < 0.001**	**F**_**5,90**_** = 10.9 P < 0.001**
CH	HSP22*	**F**_**1,90**_** = 9.3 P = 0.003**	F_2,90_ = 1.1 P = 0.330	**F**_**5,90**_** = 6.4 P < 0.001**
	Foot muscle tissue			
OS	MDA	**F**_**1,54**_** = 55.0 P < 0.001**	F_2,54_ = 2.5 P = 0.096	**F**_**5,54**_** = 4.9 P = 0.001**
OS	4-HNE	**F**_**1,54**_** = 53.2 P < 0.001**	**F**_**2,54**_** = 12.5 P < 0.001**	**F**_**5,54**_** = 9.4 P < 0.001**
OS	PC	**F**_**1,54**_** = 32.2 P < 0.001**	F_2,54_ = 1.0 P = 0.373	F_5,54_ = 1.0 P = 0.438
PS	eIF-2α	**F**_**1,37**_** = 10.5 P = 0.003**	F_2,37_ = 10.4 P = 0.001	**F**_**5,37**_** = 4.7 P = 0.003**
PS	p-eIF-2α	**F**_**1,38**_** = 17.0 P <** **0.001**	**F**_**2,38**_** = 22.6 P < 0.001**	**F**_**5,38**_** = 23.5 P < 0.001**
EM	AMPK	**F**_**1,39**_** = 146.3 P < 0.001**	**F**_**2,39**_** = 13.1 P < 0.001**	**F**_**5,39**_** = 7.7 P < 0.001**
EM	HIF-1 α	**F**_**1,34**_** = 18.0 P < 0.001**	**F**_**2,34**_** = 14.3 P < 0.001**	**F**_**5,34**_** = 28.8 P < 0.001**
PD	Ubiquitin	F_1,54_ = 0.04 P = 0.837	F_2,54_ = 0.74 P = 0.484	**F**_**5,54**_** = 4.7 P = 0.002**
	Digestive gland			
EM	PK	**F**_**1,90**_** = 18.7 P < 0.0001**	F_2,90_ = 1.6 P = 0.1667	F_5,90_ = 1.06 P = 0.4081
EM	PEPCK	F_1,90_ = 0.002 P = 0.966	F_2,90_ = 0.6 P = 0.7100	F_5,90_ = 1.7 P = 0.0784
EM	LDH	**F**_**1,90**_** = 8.212 P = 0.0048**	F_2,90_ = 0.1 P = 0.5494	F_5,90_ = 1.5 P = 0.1574
EM	PK/PEPCK	**F**_**1,90**_** = 5.7 P = 0.0187**	F_2,90_ = 1.4 P = 0.2164	**F**_**5,90**_** = 2.0 P = 0.0332**

Biomarkers for oxidative stress (OS), molecular chaperones (CH), protein synthesis (PS), protein degradation (PD) and energy metabolism (EM) were assessed. Significant effects (p < 0.05) are shown in bold. * - analyses were conducted on log-transformed data.

**Table 2:** Primer sequences and qRT-PCR conditions for target genes in *C. fluminea*.

Target	Accession number	Primer sequence
HSP70	KJ461738	HSP70-FW 5′-GAT GCC AGC GTA CAG TCC-3′
		HSP70-Rev 5′-ACA GCA TTT GTC ACT GTC TTT-3′
HSC70	KC979064	HSC70-FW 5′-TCG ATG ACA CAA ATG TTC AAA A-3′
		HSC70-Rev 5′-ACA GCG TTT TTA ATC TTT TGA C-3′
HSP60	KC979065	HSP60-FW 5′-GGG ACA ACA GAA AGA ACA CCC-3′
		HSP60-Rev 5′-CCG ACA CGA CCA AAG TCA TT-3′
HSP90	KC979063	HSP90-FW 5′-GCA GCG AAG AAA CAC CTA GA-3′
		HSP90-Rev 5′-TGG GTC TTC CAG AGC AAA TC-3′
HSP40	KF218339	HSP40-FW 5′-GGT GAC CAA GGA CCC AAT AA-3′
		HSP40-Rev 5′-ACA TCC AGT CAG AGC CTT TC-3′
HSP22	KF218338	HSP22-FW 5′-GGA ATT CGA GGA GTC GAG AAA-3′
		HSP22-Rev 5′-CTT TCA ACC TGG AGC ATT GTG-3′
GSTS1	KJ001775	GSTS1-FW 5′-TGG GCC TGG TGG AAG ATA-3′
		GSTS1-Rev 5′-ACA AGT CGG CCA ATG TGA G-3′
GSTM1	KJ001774	GSTM1-FW 5′-GTC CCA ACC TTC ATG TTC CT-3′
		GSTM1-Rev 5′-AGC CAC CAT AAC GTC TCT TG-3′
GSTμ	KF218346	GSTμ-FW 5′-ACG CAG AGT TCC TTG ACT TT-3′
		GSTμ-Rev 5′-GGC CTT ATC CCT CTC TGT TTC-3′
TXNRD1	KF275126	TXNRD1-FW 5′-CGC TTG TAG TAG GTG CAT CA-3′
		TXNRD1- Rev 5′-GTC AAA TCC TCG CAG GAG AA-3′
TXNIP	KF275125	TXNIP-FW 5′-ACA GAG GTG CCT GAC TTT AAC-3′
		TXNIP - Rev 5′-ACC TGT ACC CAA GTC AAT CAT-3′
TPX1	KF218348	TPX1-FW 5′-TCT GTC CGA CTG AAG TTG TG-3′
		TPX1-Rev 5′-CCA CTG GAT CTT GGA GTG TT-3′
Cu,Zn-SOD	KF218347	Cu,Zn-SOD-FW 5′-GCA GGA GCT CAC TAC AAT CC-3′
		Cu,Zn-SOD-Rev 5′-CAC CTT ACA TCC AGC CTC ATC-3′
Mn-SOD	EF446611	Mn-SOD-FW 5′-CAG GCT AAT GGC AGA CTT CA-3′
		Mn-SOD-Rev 5′-GTA GTA AGC GTG CTC CCA AA-3′
GPxA	KF218345	GPxA-FW 5′-GTT CTC TCA CCC AGC TGA TTA C-3′
		GPxA-Rev 5′-GTG CCT GCA TAG TCC AAG AT-3′
MT	EF185126	MT-FW 5′-TGC AAG TGA TGG GCT AAA GT-3′
		MT-Rev 5′-GAC TTA CAG CGG TGC ATA GAA-3′
CAT	EF446609	CAT-FW 5′-ACG GTA GCC ACA CAT TCA AG-3′
		CAT-Rev 5′-GGT AGT TGC CCT CAG CAA TAG-3′
β-actin	EF446608	Act-FW 5′-TAC CAC AAC AGC CGA AAG AG-3′
		Act-Rev 5′-GAA TGA GGG CTG GAG CAT AG-3′
RSP9	AY571758	RSP9-FW 5′-CGT GAA GTG TGG AGA GTC AAG-3′
		RSP9-Rev 5′-ACC AAA CGA CGC AGA AGA G-3′

Abbreviations: HSP70, inducible heat shock protein 70; HSC70, constitutive heat shock protein 70; HSP60, heat shock protein 60; HSP90, heat shock protein 90; HSP40, heat shock protein 40, HSP22, heat shock protein 22; TXNRD1, thioredoxin reductase 1; TXNIP, thioredoxin-interacting protein; TPX1, thioredoxin peroxidase; Cu,Zn-SOD, Cu,Zn-superoxide dismutase; Mn-SOD, Mn- superoxide dismutase; GSTS1, glutathione S-transferase sigma 1; GSTM1, glutathione S-transferase μ1; GSTμ, glutathione *S*-transferase μ; GPxA, *glutathione* peroxidase; MT, metallothionein; CAT, catalase; RSP9, 40 S ribosomal protein S9.
