# Supplementary material for: Long-Term Acclimation to Different Thermal Regimes Affects Molecular Responses to Heat Stress in a Freshwater Clam Corbicula Fluminea
Source: Sci Rep. 2016 Dec 20;6:39476. doi: 10.1038/srep39476 (PMC5171794; doi:10.1038/srep39476)
Supplement: Supplementary Information [file srep39476-s1.pdf]

Supplementary information to the manuscript "LONG-TERM ACCLIMATION TO DIFFERENT THERMAL REGIMES AFFECTS MOLECULAR RESPONSES TO HEAT STRESS IN A FRESHWATER CLAM *CORBICULA FLUMINEA*" by Halina I. Falfushynska, Tuan Phan, and Inna M. Sokolova

Supplementary Figure 1. Representative images of Western Blot (A-D) and dot-blot (E) analyses of tissue extracts from *C. fluminea* using eiF2α (A), p-eiF2α (B), AMPKα (C), HIF-1α (D), and ubiquitin (E) antibodies. The first lane on images A-D represents a molecular weight marker. Western blot for HIF-1α (D) yielded two bands corresponding to the intact HIF-1α (~100 kDa) and its degradation products (the lower band of ~70 kDa); only the upper band was quantified.

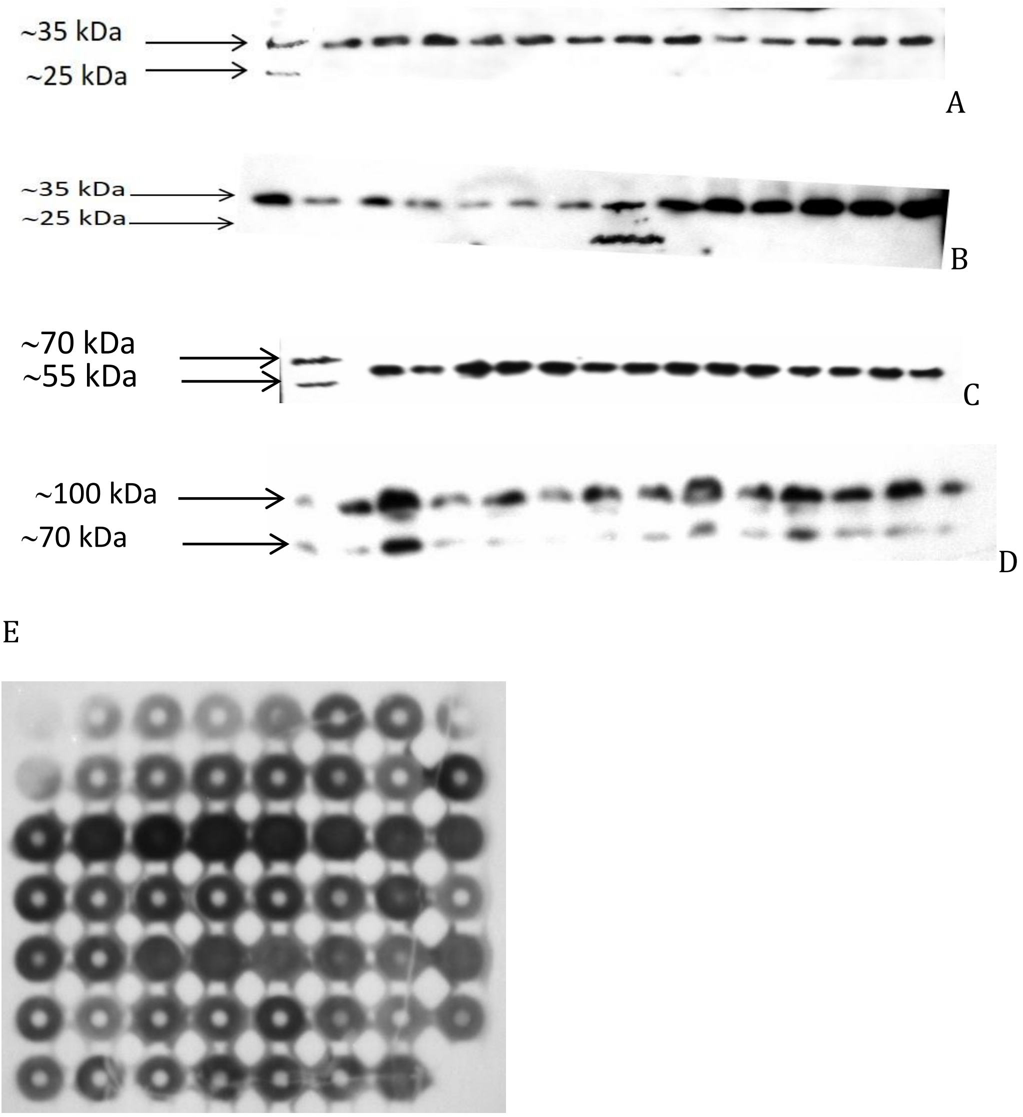

Supplementary Table 1. Pearson correlations among the studied traits across four temperature exposure groups (20, 26, 32 and 38°C) in the three studied populations of *C. fluminea*. N=91 with each replicate representing an individual clam. Significant correlations are highlighted in red.

	MTs	Mn-SOD	GSTμ	TPX1	Cu,Zn-SOD	GPxA	GSTM1	TXNRD1	CAT	HSC70	TXNLP	GSTS1	HSP60	HSP40	HSP70	HSP90	HSP22	MDA	4-HNE	PC	eiF-2a	AMPK	p-eiF2a	HIF-1a	Ubiquitin	PK	PEPCK	LDH
**MTs**	1.0000	-.0954	.0060	.3775	.1985	.1118	.2522	.1782	-.0673	.2343	.2723	.2343	.4720	-.2362	.5406	.4110	.6914	.5184	-.2546	.2271	.3508	.1119	-.0559	.3600	.1815	.0526	.3733	.2380
	p= ---	p=.368	p=.955	p=.000	p=.059	p=.291	p=.016	p=.091	p=.526	p=.025	p=.009	p=.025	p=.000	p=.024	p=.000	p=.000	p=.000	p=.000	p=.015	p=.030	p=.001	p=.291	p=.598	p=.000	p=.085	p=.621	p=.000	p=.023
**Mn-SOD**	-.0954	1.0000	.5637	.2748	.3232	.5149	.1739	.3953	.4576	-.0098	-.1726	-.0098	-.2409	.4253	-.0511	-.0645	-.0430	.0010	.1732	-.0653	-.2244	-.1766	-.0797	.2654	-.1809	.0408	-.1545	.0547
	p=.368	p= ---	p=.000	p=.008	p=.002	p=.000	p=.099	p=.000	p=.000	p=.927	p=.102	p=.927	p=.021	p=.000	p=.630	p=.544	p=.686	p=.993	p=.101	p=.538	p=.033	p=.094	p=.453	p=.011	p=.086	p=.701	p=.144	p=.606
**GSTμ**	.0060	.5637	1.0000	.3302	.3542	.5599	.1832	.0629	.5912	.0189	-.0841	.0189	-.2750	.2776	.0733	.0957	.0488	.1592	.2207	.1280	-.3233	-.2962	-.2300	.3360	-.0473	.1129	-.0638	.1345
	p=.955	p=.000	p= ---	p=.001	p=.001	p=.000	p=.082	p=.554	p=.000	p=.859	p=.428	p=.859	p=.008	p=.008	p=.490	p=.367	p=.646	p=.132	p=.036	p=.227	p=.002	p=.004	p=.028	p=.001	p=.656	p=.287	p=.548	p=.204
**TPX1**	.3775	.2748	.3302	1.0000	.2425	.3915	.2295	.0674	.0224	-.0087	-.0068	-.0087	.2356	.0322	.4817	.3449	.3713	.4328	.2021	.2733	.1030	-.2551	.0546	.3626	-.0382	.1292	.0499	.0693
	p=.000	p=.008	p=.001	p= ---	p=.021	p=.000	p=.029	p=.525	p=.833	p=.935	p=.949	p=.935	p=.025	p=.762	p=.000	p=.001	p=.000	p=.000	p=.055	p=.009	p=.332	p=.015	p=.607	p=.000	p=.719	p=.222	p=.639	p=.514
**Cu,Zn-SOD**	.1985	.3232	.3542	.2425	1.0000	.4809	.6442	.1140	.4875	.1688	.0154	.1688	.0561	.2525	.1894	.1390	.0520	-.0273	-.1829	-.1257	-.0020	.1422	.0347	.0427	-.0130	-.0642	-.0309	.0703
	p=.059	p=.002	p=.001	p=.021	p= ---	p=.000	p=.000	p=.282	p=.000	p=.110	p=.885	p=.110	p=.597	p=.016	p=.072	p=.189	p=.624	p=.797	p=.083	p=.235	p=.985	p=.179	p=.744	p=.688	p=.903	p=.545	p=.771	p=.508
**GPxA**	.1118	.5149	.5599	.3915	.4809	1.0000	.3806	.1199	.3915	.1383	.0105	.1383	-.0740	.3497	.3853	.3662	.0372	.2179	.2015	.1698	-.0791	-.1476	-.0935	.2340	-.1650	.0740	-.0092	.2012
	p=.291	p=.000	p=.000	p=.000	p=.000	p= ---	p=.000	p=.258	p=.000	p=.191	p=.921	p=.191	p=.486	p=.001	p=.000	p=.000	p=.726	p=.038	p=.056	p=.108	p=.456	p=.163	p=.378	p=.026	p=.118	p=.486	p=.931	p=.056
**GSTM1**	.2522	.1739	.1832	.2295	.6442	.3806	1.0000	.0491	.3962	.2696	.1521	.2696	.1757	.1059	.0076	.0131	.0419	-.0940	-.3441	-.2636	-.2124	-.0274	.0274	-.1179	-.0026	-.0161	-.0209	-.0070
	p=.016	p=.099	p=.082	p=.029	p=.000	p=.000	p= ---	p=.644	p=.000	p=.010	p=.150	p=.010	p=.096	p=.318	p=.943	p=.902	p=.693	p=.376	p=.001	p=.012	p=.043	p=.797	p=.796	p=.266	p=.980	p=.879	p=.844	p=.947
**TXNRD1**	.1782	.3953	.0629	.0674	.1140	.1199	.0491	1.0000	.0401	.5356	.4098	.5356	.4267	-.0169	.1252	.0391	.0120	.0732	-.0372	-.1574	.0946	-.0597	.0613	.3210	.0322	.0145	-.0327	.0548
	p=.091	p=.000	p=.554	p=.525	p=.282	p=.258	p=.644	p= ---	p=.706	p=.000	p=.000	p=.000	p=.000	p=.873	p=.237	p=.713	p=.910	p=.491	p=.726	p=.136	p=.372	p=.574	p=.563	p=.002	p=.762	p=.892	p=.758	p=.606
**CAT**	-.0673	.4576	.5912	.0224	.4875	.3915	.3962	.0401	1.0000	.1110	-.0705	.1110	-.2807	.3120	-.1920	-.1592	-.2073	-.2569	-.1705	-.1782	-.3624	.0070	-.0488	-.0910	-.1320	.0324	.0306	.1160
	p=.526	p=.000	p=.000	p=.833	p=.000	p=.000	p=.000	p=.706	p= ---	p=.295	p=.507	p=.295	p=.007	p=.003	p=.068	p=.132	p=.049	p=.014	p=.106	p=.091	p=.000	p=.948	p=.646	p=.391	p=.212	p=.760	p=.773	p=.274
**HSC70**	.2343	-.0098	.0189	-.0087	.1688	.1383	.2696	.5356	.1110	1.0000	.6490	1.0000	.4142	-.0295	.0947	.0916	-.1180	-.0738	-.1641	-.2336	.1290	.1355	.1003	-.0027	.0631	-.0855	.0150	-.0106
	p=.025	p=.927	p=.859	p=.935	p=.110	p=.191	p=.010	p=.000	p=.295	p= ---	p=.000	p= ---	p=.000	p=.781	p=.372	p=.388	p=.265	p=.487	p=.120	p=.026	p=.223	p=.200	p=.344	p=.979	p=.552	p=.420	p=.888	p=.921
**TXNLP**	.2723	-.1726	-.0841	-.0068	.0154	.0105	.1521	.4098	-.0705	.6490	1.0000	.6490	.3399	-.1724	.0862	.0548	-.0143	.0343	-.3397	-.0925	.0704	.0510	.1448	-.0116	.0339	-.0349	.1289	.0880
	p=.009	p=.102	p=.428	p=.949	p=.885	p=.921	p=.150	p=.000	p=.507	p=.000	p= ---	p=.000	p=.001	p=.102	p=.417	p=.606	p=.893	p=.747	p=.001	p=.383	p=.507	p=.631	p=.171	p=.913	p=.750	p=.743	p=.223	p=.407
**GSTS1**	.2343	-.0098	.0189	-.0087	.1688	.1383	.2696	.5356	.1110	1.0000	.6490	1.0000	.4142	-.0295	.0947	.0916	-.1180	-.0738	-.1641	-.2336	.1290	.1355	.1003	-.0027	.0631	-.0855	.0150	-.0106
	p=.025	p=.927	p=.859	p=.935	p=.110	p=.191	p=.010	p=.000	p=.295	p= ---	p=.000	p= ---	p=.000	p=.781	p=.372	p=.388	p=.265	p=.487	p=.120	p=.026	p=.223	p=.200	p=.344	p=.979	p=.552	p=.420	p=.888	p=.921
**HSP60**	.4720	-.2409	-.2750	.2356	.0561	-.0740	.1757	.4267	-.2807	.4142	.3399	.4142	1.0000	-.2719	.3962	.3002	.2037	.2457	-.0493	-.0033	.3511	.0848	.0836	.1974	.2245	-.0022	-.0039	-.0047
	p=.000	p=.021	p=.008	p=.025	p=.597	p=.486	p=.096	p=.000	p=.007	p=.000	p=.001	p=.000	p= ---	p=.009	p=.000	p=.004	p=.053	p=.019	p=.642	p=.975	p=.001	p=.424	p=.431	p=.061	p=.032	p=.984	p=.971	p=.964
**HSP40**	-.2362	.4253	.2776	.0322	.2525	.3497	.1059	-.0169	.3120	-.0295	-.1724	-.0295	-.2719	1.0000	-.2106	-.1442	-.1477	-.2206	.2684	-.1552	-.0340	.1276	.1313	-.1089	-.2666	-.1285	-.1143	-.1827
	p=.024	p=.000	p=.008	p=.762	p=.016	p=.001	p=.318	p=.873	p=.003	p=.781	p=.102	p=.781	p=.009	p= ---	p=.045	p=.173	p=.162	p=.036	p=.010	p=.142	p=.749	p=.228	p=.215	p=.304	p=.011	p=.225	p=.281	p=.083
**HSP70**	.5406	-.0511	.0733	.4817	.1894	.3853	.0076	.1252	-.1920	.0947	.0862	.0947	.3962	-.2106	1.0000	.8891	.4296	.7001	.1209	.4573	.3448	-.0117	-.1731	.5172	.1019	.1330	.1341	.2366
	p=.000	p=.630	p=.490	p=.000	p=.072	p=.000	p=.943	p=.237	p=.068	p=.372	p=.417	p=.372	p=.000	p=.045	p= ---	p=0.00	p=.000	p=.000	p=.254	p=.000	p=.001	p=.912	p=.101	p=.000	p=.337	p=.209	p=.205	p=.024
**HSP90**	.4110	-.0645	.0957	.3449	.1390	.3662	.0131	.0391	-.1592	.0916	.0548	.0916	.3002	-.1442	.8891	1.0000	.2853	.6085	.1681	.4457	.2740	-.0085	-.1619	.4014	.0579	.1441	.0822	.3045
	p=.000	p=.544	p=.367	p=.001	p=.189	p=.000	p=.902	p=.713	p=.132	p=.388	p=.606	p=.388	p=.004	p=.173	p=0.00	p= ---	p=.006	p=.000	p=.111	p=.000	p=.009	p=.937	p=.125	p=.000	p=.585	p=.173	p=.438	p=.003
**HSP22**	.6914	-.0430	.0488	.3713	.0520	.0372	.0419	.0120	-.2073	-.1180	-.0143	-.1180	.2037	-.1477	.4296	.2853	1.0000	.6444	.0625	.3083	.2165	-.0199	-.1839	.4639	.1908	.0930	.4542	.2772
	p=.000	p=.686	p=.646	p=.000	p=.624	p=.726	p=.693	p=.910	p=.049	p=.265	p=.893	p=.265	p=.053	p=.162	p=.000	p=.006	p= ---	p=.000	p=.556	p=.003	p=.039	p=.851	p=.081	p=.000	p=.070	p=.381	p=.000	p=.008
**MDA**	.5184	.0010	.1592	.4328	-.0273	.2179	-.0940	.0732	-.2569	-.0738	.0343	-.0738	.2457	-.2206	.7001	.6085	.6444	1.0000	.2527	.6302	.1688	-.2558	-.4187	.5936	.1822	.1792	.1937	.3034
	p=.000	p=.993	p=.132	p=.000	p=.797	p=.038	p=.376	p=.491	p=.014	p=.487	p=.747	p=.487	p=.019	p=.036	p=.000	p=.000	p=.000	p= ---	p=.016	p=.000	p=.110	p=.014	p=.000	p=.000	p=.084	p=.089	p=.066	p=.003
**4-HNE**	-.2546	.1732	.2207	.2021	-.1829	.2015	-.3441	-.0372	-.1705	-.1641	-.3397	-.1641	-.0493	.2684	.1209	.1681	.0625	.2527	1.0000	.3309	.0785	-.2438	-.0455	.4070	-.2330	.1220	-.2234	.0661

	p=.015	p=.101	p=.036	p=.055	p=.083	p=.056	p=.001	p=.726	p=.106	p=.120	p=.001	p=.120	p=.642	p=.010	p=.254	p=.111	p=.556	p=.016	p= ---	**p=.001**	p=.460	**p=.020**	p=.669	**p=.000**	**p=.026**	p=.249	**p=.033**	p=.533
**PC**	.2271	-.0653	.1280	.2733	-.1257	.1698	-.2636	-.1574	-.1782	-.2336	-.0925	-.2336	-.0033	-.1552	.4573	.4457	.3083	.6302	.3309	1.0000	.0544	**-.3726**	**-.2512**	**.3711**	.0582	**.2177**	.1624	**.2518**
	p=.030	p=.538	p=.227	p=.009	p=.235	p=.108	p=.012	p=.136	p=.091	p=.026	p=.383	p=.026	p=.975	p=.142	p=.000	p=.000	p=.003	p=.000	p=.001	p= ---	p=.608	**p=.000**	**p=.016**	**p=.000**	p=.584	**p=.038**	p=.124	**p=.016**
**eiF-2a**	.3508	-.2244	-.3233	.1030	-.0020	-.0791	-.2124	.0946	-.3624	.1290	.0704	.1290	.3511	-.0340	.3448	.2740	.2165	.1688	.0785	.0544	1.0000	**.5301**	**.3012**	.1534	-.0023	-.0978	.1644	-.0583
	p=.001	p=.033	p=.002	p=.332	p=.985	p=.456	p=.043	p=.372	p=.000	p=.223	p=.507	p=.223	p=.001	p=.749	p=.001	p=.009	p=.039	p=.110	p=.460	p=.608	p= ---	**p=.000**	**p=.004**	p=.147	p=.983	p=.357	p=.119	p=.583
**AMPK**	.1119	-.1766	-.2962	-.2551	.1422	-.1476	-.0274	-.0597	.0070	.1355	.0510	.1355	.0848	.1276	-.0117	-.0085	-.0199	-.2558	-.2438	-.3726	.5301	1.0000	.0592	-.1054	.0269	**-.2143**	**.2080**	-.1252
	p=.291	p=.094	p=.004	p=.015	p=.179	p=.163	p=.797	p=.574	p=.948	p=.200	p=.631	p=.200	p=.424	p=.228	p=.912	p=.937	p=.851	p=.014	p=.020	p=.000	p=.000	p= ---	p=.578	p=.320	p=.800	**p=.041**	**p=.048**	p=.237
**p-eiF2a**	-.0559	-.0797	-.2300	.0546	.0347	-.0935	.0274	.0613	-.0488	.1003	.1448	.1003	.0836	.1313	-.1731	-.1619	-.1839	-.4187	-.0455	-.2512	.3012	.0592	1.0000	**-.3433**	**-.3680**	-.1876	-.0209	-.0912
	p=.598	p=.453	p=.028	p=.607	p=.744	p=.378	p=.796	p=.563	p=.646	p=.344	p=.171	p=.344	p=.431	p=.215	p=.101	p=.125	p=.081	p=.000	p=.669	p=.016	p=.004	p=.578	p= ---	**p=.001**	**p=.000**	p=.075	p=.844	p=.390
**HIF-1a**	.3600	.2654	.3360	.3626	.0427	.2340	-.1179	.3210	-.0910	-.0027	-.0116	-.0027	.1974	-.1089	.5172	.4014	.4639	.5936	.4070	.3711	.1534	-.1054	-.3433	1.0000	.1098	.1655	.0544	**.2859**
	p=.000	p=.011	p=.001	p=.000	p=.688	p=.026	p=.266	p=.002	p=.391	p=.979	p=.913	p=.979	p=.061	p=.304	p=.000	p=.000	p=.000	p=.000	p=.000	p=.000	p=.147	p=.320	p=.001	p= ---	p=.300	p=.117	p=.609	**p=.006**
**Ubiquitin**	.1815	-.1809	-.0473	-.0382	-.0130	-.1650	-.0026	.0322	-.1320	.0631	.0339	.0631	.2245	-.2666	.1019	.0579	.1908	.1822	-.2330	.0582	-.0023	.0269	-.3680	.1098	1.0000	.1434	.1247	.1125
	p=.085	p=.086	p=.656	p=.719	p=.903	p=.118	p=.980	p=.762	p=.212	p=.552	p=.750	p=.552	p=.032	p=.011	p=.337	p=.585	p=.070	p=.084	p=.026	p=.584	p=.983	p=.800	p=.000	p=.300	p= ---	p=.175	p=.239	p=.289
**PK**	.0526	.0408	.1129	.1292	-.0642	.0740	-.0161	.0145	.0324	-.0855	-.0349	-.0855	-.0022	-.1285	.1330	.1441	.0930	.1792	.1220	.2177	-.0978	-.2143	-.1876	.1655	.1434	1.0000	.1677	**.4047**
	p=.621	p=.701	p=.287	p=.222	p=.545	p=.486	p=.879	p=.892	p=.760	p=.420	p=.743	p=.420	p=.984	p=.225	p=.209	p=.173	p=.381	p=.089	p=.249	p=.038	p=.357	p=.041	p=.075	p=.117	p=.175	p= ---	p=.112	**p=.000**
**PEPCK**	.3733	-.1545	-.0638	.0499	-.0309	-.0092	-.0209	-.0327	.0306	.0150	.1289	.0150	-.0039	-.1143	.1341	.0822	.4542	.1937	-.2234	.1624	.1644	.2080	-.0209	.0544	.1247	.1677	1.0000	**.4167**
	p=.000	p=.144	p=.548	p=.639	p=.771	p=.931	p=.844	p=.758	p=.773	p=.888	p=.223	p=.888	p=.971	p=.281	p=.205	p=.438	p=.000	p=.066	p=.033	p=.124	p=.119	p=.048	p=.844	p=.609	p=.239	p=.112	p= ---	**p=.000**
**LDH**	.2380	.0547	.1345	.0693	.0703	.2012	-.0070	.0548	.1160	-.0106	.0880	-.0106	-.0047	-.1827	.2366	.3045	.2772	.3034	.0661	.2518	-.0583	-.1252	-.0912	.2859	.1125	.4047	.4167	1.0000
	p=.023	p=.606	p=.204	p=.514	p=.508	p=.056	p=.947	p=.606	p=.274	p=.921	p=.407	p=.921	p=.964	p=.083	p=.024	p=.003	p=.008	p=.003	p=.533	p=.016	p=.583	p=.237	p=.390	p=.006	p=.289	p=.000	p=.000	p= ---
